# Supplementary material for: A Combination of Pharmacophore-Based Virtual Screening, Structure-Based Lead Optimization, and DFT Study for the Identification of S. epidermidis TcaR Inhibitors
Source: Pharmaceuticals (Basel). 2022 May 21;15(5):635. doi: 10.3390/ph15050635 (PMC9146354; doi:10.3390/ph15050635)
Supplement: Supplementary file 1 [file pharmaceuticals-15-00635-s001.zip › pharmaceuticals-1705007-supplementary.pdf]

Supplementary Material

A Combination of Pharmacophore-based Virtual Screening, Structure-based Lead Optimization and DFT/Quantum Mechanics for the Identification of S. epidermidis TcaR inhibitors

Srimai Vuppala[1], Jaeyoung Kim[1], Bo Sun Joo[2*], Ji Myung Choi[3], and Joonkyung Jang[1]*

[1]Department of Nanoenergy Engineering, Pusan National University, Busan 46241, Republic of Korea.

[2]Infertility Institute, Pohang Women's Hospital, Pohang 37754, Republic of Korea

[3]Lab-to-Medi CRO, 12 Dosandae-ro-8-gil, Seoul 06038 Republic of Korea

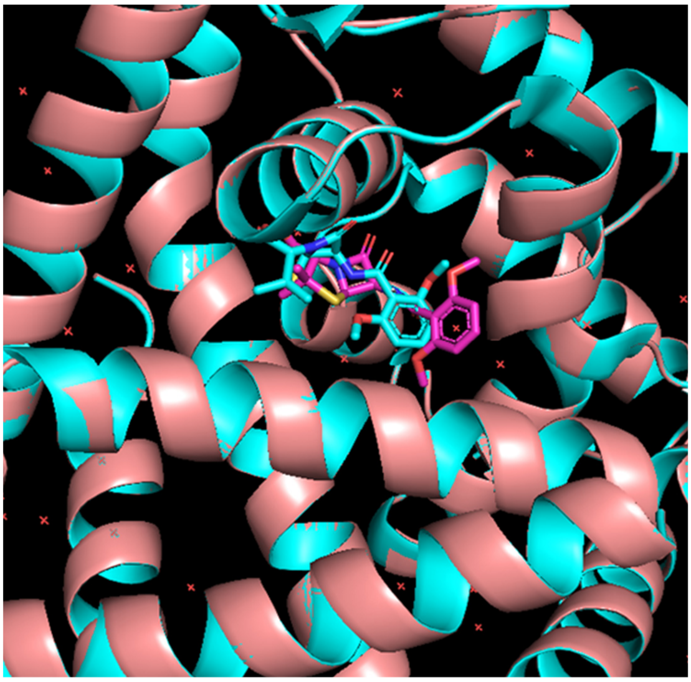

Figure S1. Superimposition of docked conformation of methicillin (purple color)-TcaR (red color) complex with crystal structure (PDB ID 3KP4) conformation (blue color).

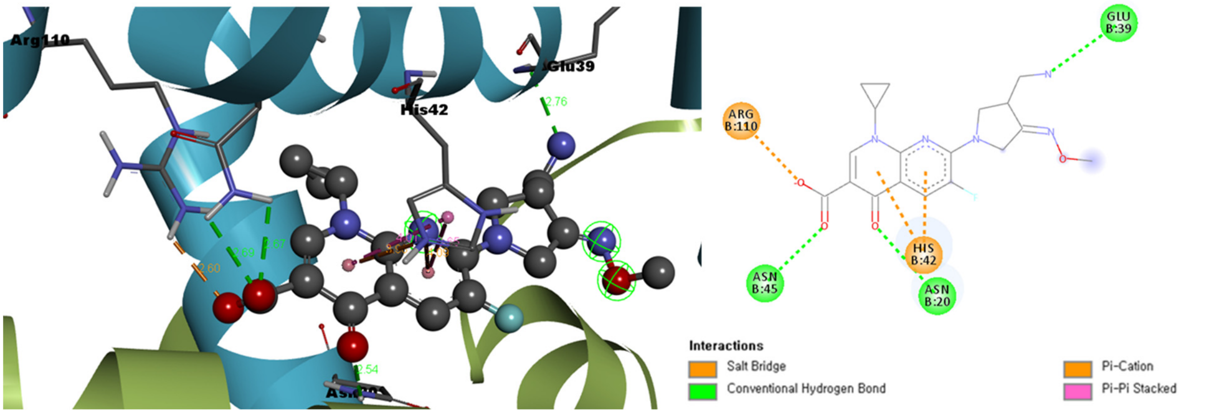

Figure S2. The binding mode conformation and molecular interactions of gemifloxacin in the active site of *S. epidermidis* TcaR. The left and right figures represent the molecular interactions in 3D and 2D, respectively. The hit compound is shown in the ball-stick model, whereas the key interacting residues are shown as grey sticks.

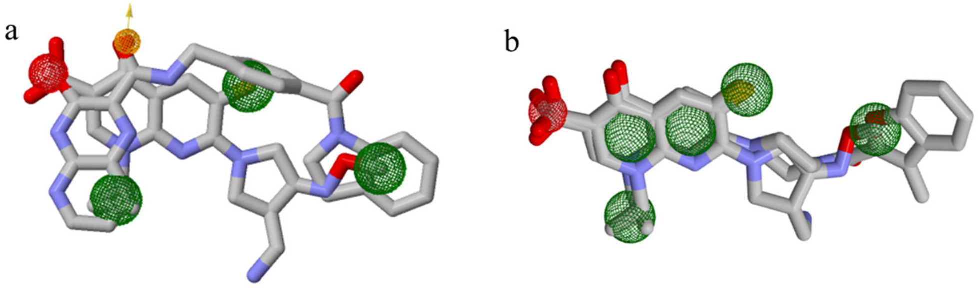

Figure S3. Diagrams of **a** and **b** represent the overlay of pharmacophoric features of the first and second models with their hit compounds ZINC77906236, and ZINC09550296, respectively.

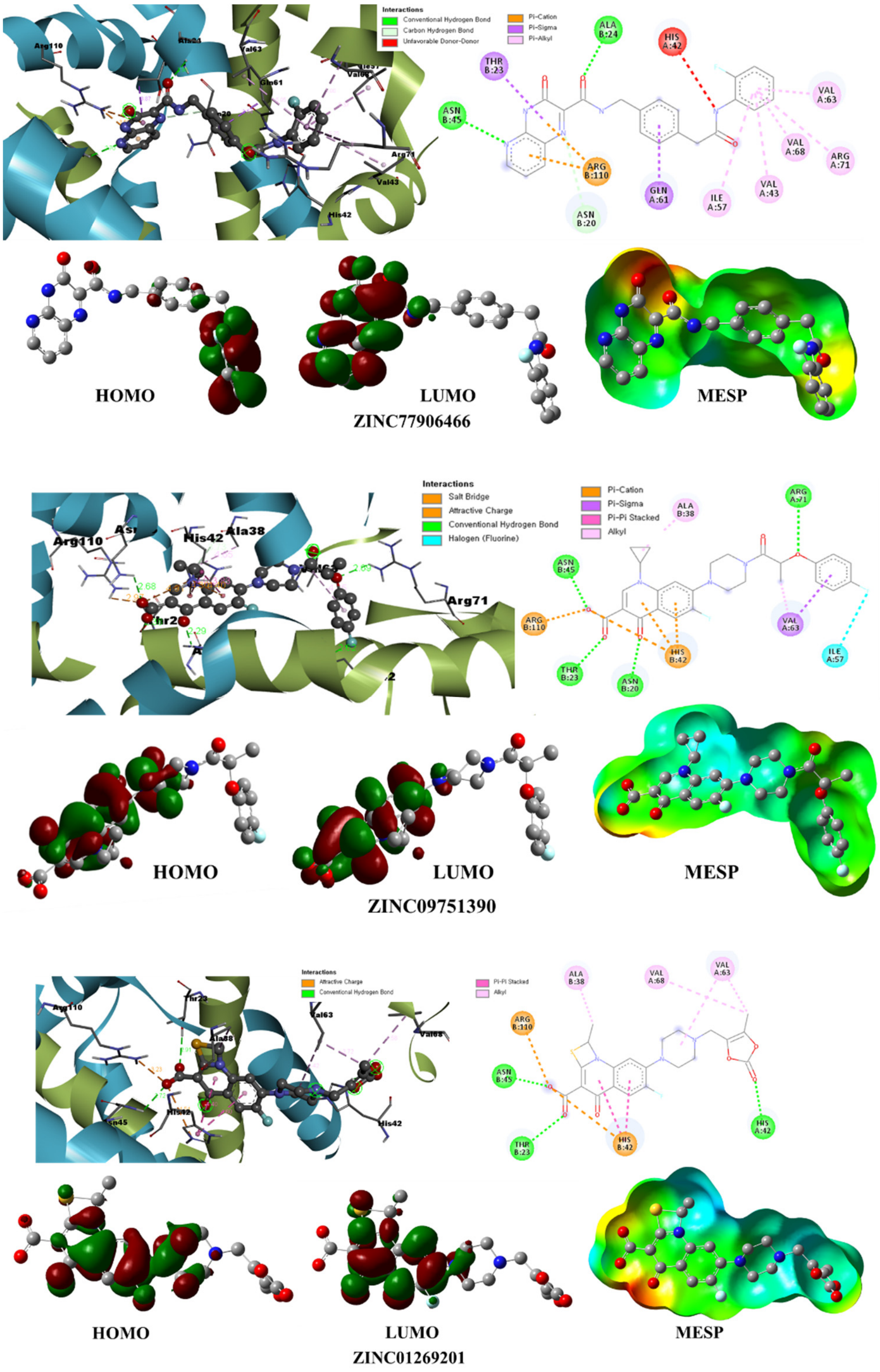

Figure S4. Binding mode conformations and molecular interactions of hit compounds ZINC77906466, ZINC09751390, and ZINC01269201 in the active site of *S. epidermidis* TcaR. The left and right figures represent the molecular interactions in 3D and 2D, respectively. The hit compounds are shown in the ball-stick model and the key interacting residues are shown as grey sticks. Isodensity plots of HOMO and LUMO and molecular electrostatic potential of hit compound.

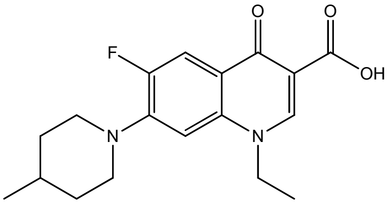

Mol1

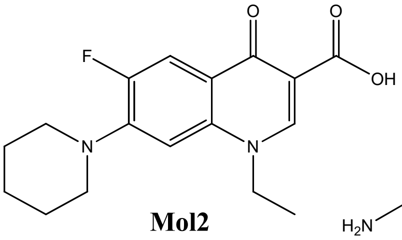

Mol2

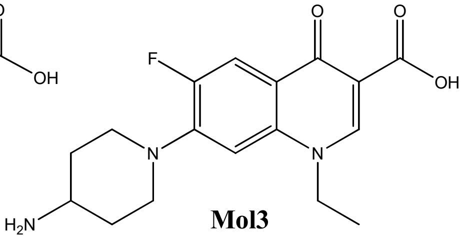

Mol3

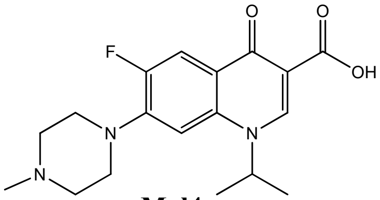

Mol4

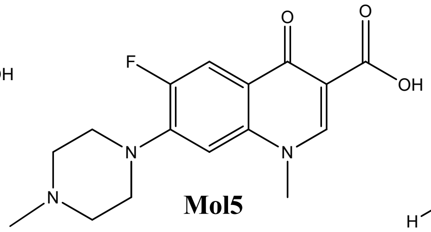

Mol5

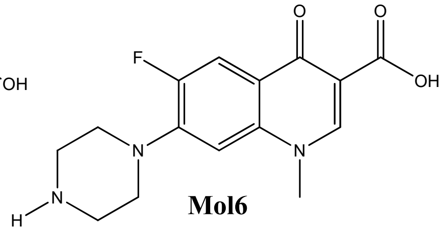

Mol6

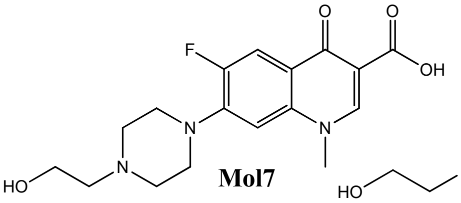

Mol7

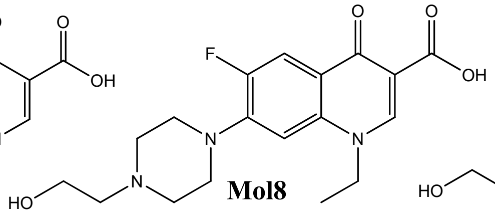

Mol8

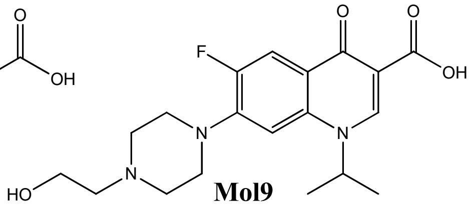

Mol9

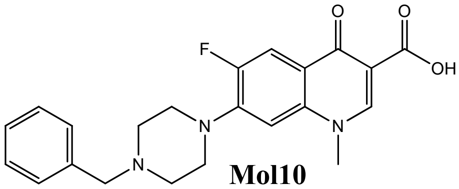

Mol10

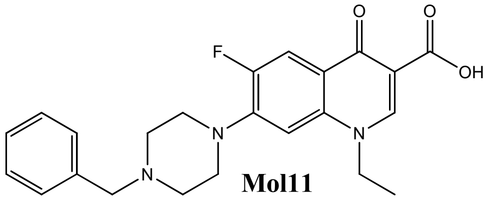

Mol11

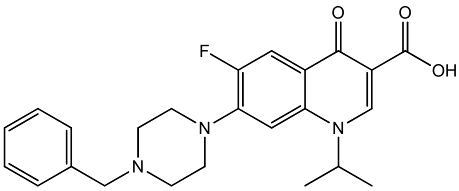

Mol12

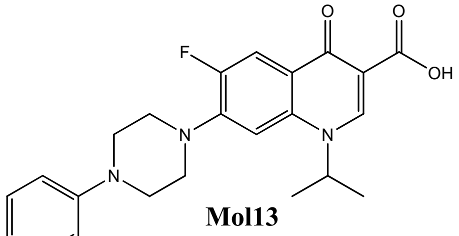

Mol13

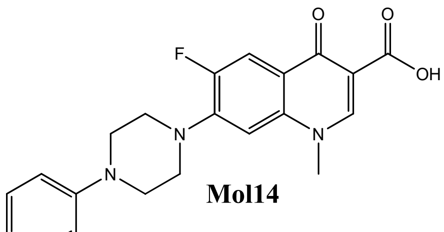

Mol14

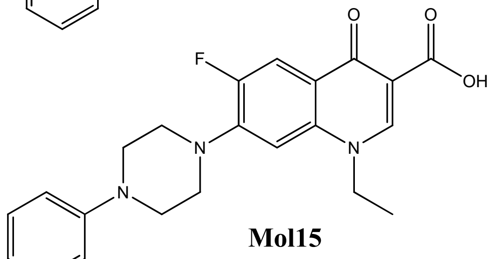

Mol15

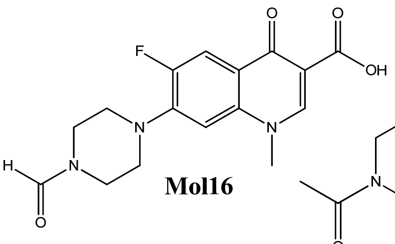

Mol16

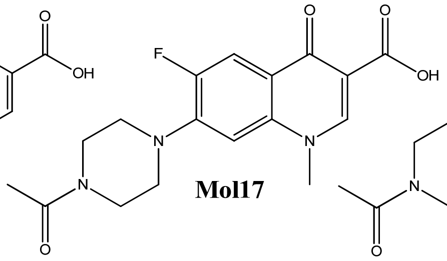

Mol17

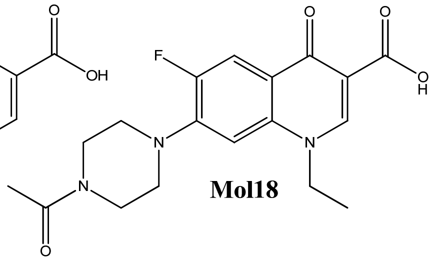

Mol18

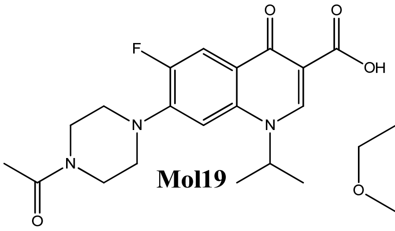

Mol19

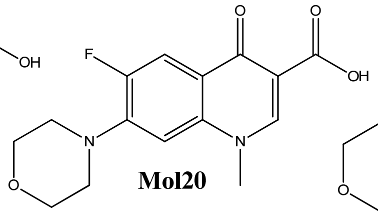

Mol20

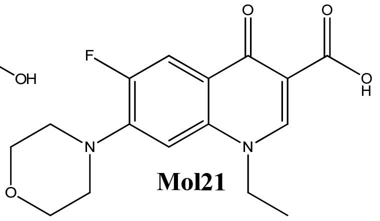

Mol21

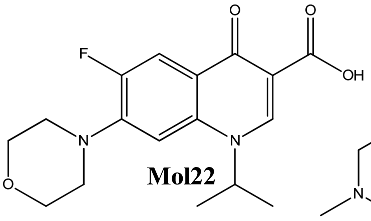

Mol22

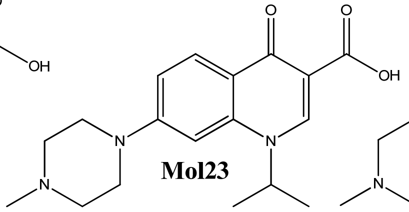

Mol23

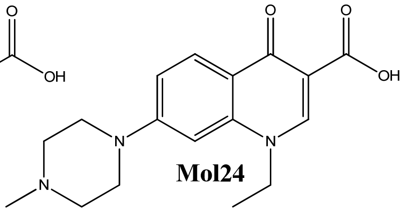

Mol24

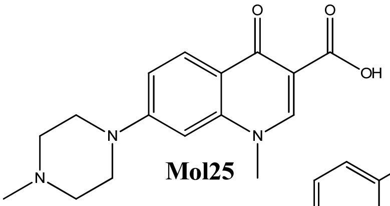

Mol25

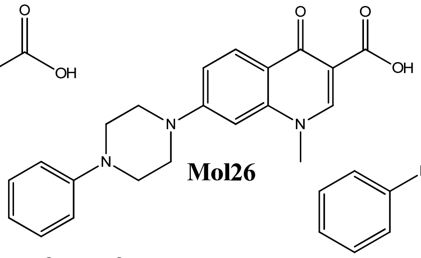

Mol26

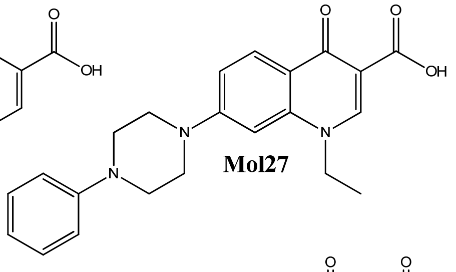

Mol27

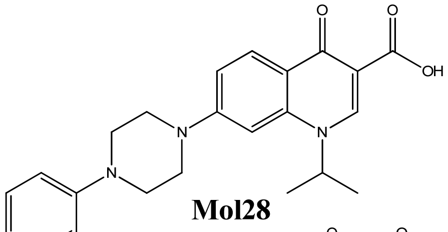

Mol28

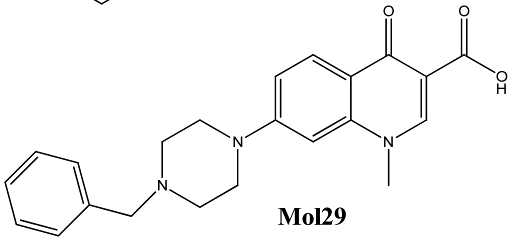

Mol29

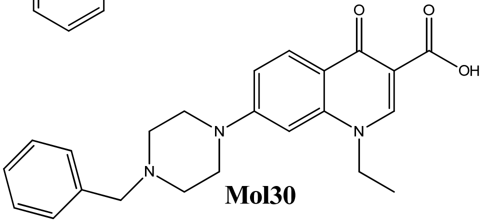

Mol30

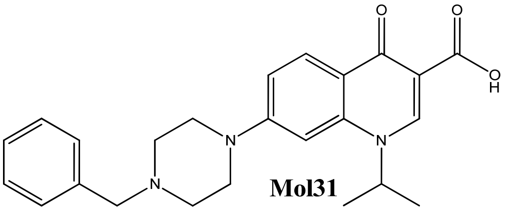

Mol31

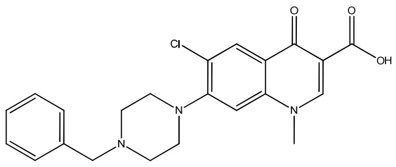

Mol32

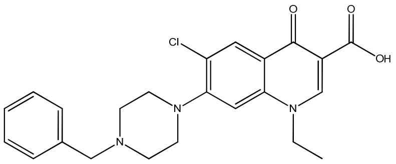

Mol33

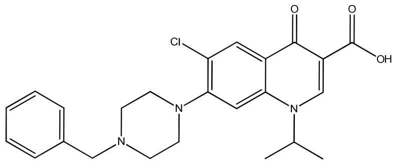

Mol34

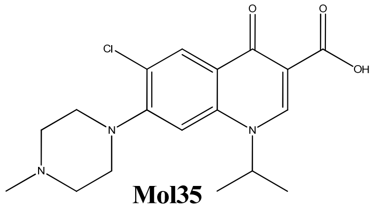

Mol35

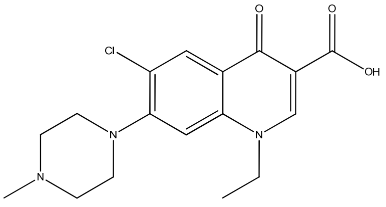

Mol36

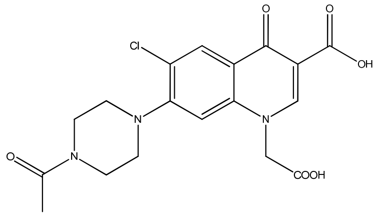

Mol37

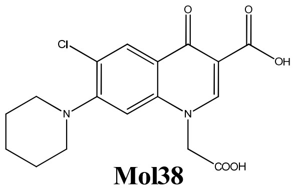

Mol38

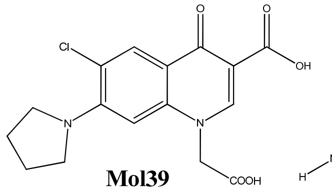

Mol39

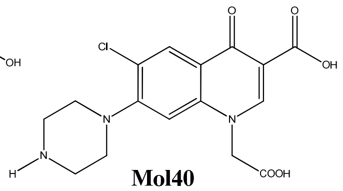

Mol40

Figure S5. Molecular structures of designed quinolone derivatives.

Table. S1 Calculated SAR properties of the dataset obtained from the Molinspiration program.

Compounds	TPSA[a]	N atoms	M.Wt[b]	nON[c]	nOHNH[d]	n violations	n rotb[e]	volume	Enzyme inhibitor
**ZINC77906236**	**108.31**	**32**	**425.45**	**8**	**8**	**0**	**4**	**369.12**	**0.09**
**ZINC03114214**	**104.45**	**32**	**449.89**	**7**	**1**	**1**	**4**	**382.66**	**-0.35**
**ZINC09550296**	**95.99**	**35**	**477.49**	**8**	**1**	**0**	**4**	**412.13**	**-0.06**
**ZINC77906466**	**117.10**	**32**	**431.43**	**8**	**3**	**0**	**6**	**367.71**	**0.08**
**ZINC01958447**	**104.45**	**31**	**456.29**	**7**	**1**	**1**	**4**	**363.07**	**-0.34**
**ZINC09751390**	**92.08**	**36**	**497.50**	**8**	**1**	**0**	**6**	**423.54**	**0.06**
**ZINC01269201**	**109.13**	**32**	**461.47**	**9**	**1**	**0**	**4**	**373.82**	**0.13**
**ZINC21985520**	**107.44**	**32**	**463.49**	**9**	**1**	**0**	**4**	**380.06**	**0.07**
**ZINC09751395**	**92.08**	**36**	**497.50**	**8**	**1**	**0**	**6**	**423.54**	**0.06**
**ZINC02280291**	**94.88**	**35**	**500.53**	**8**	**2**	**1**	**6**	**415.20**	**-0.04**
**ZINC01440193**	**94.88**	**35**	**496.56**	**8**	**2**	**0**	**6**	**426.83**	**-0.07**
**ZINC01127091**	**77.81**	**33**	**470.57**	**7**	**2**	**0**	**6**	**409.70**	**-0.01**
**ZINC72332562**	**100.47**	**31**	**429.50**	**7**	**2**	**1**	**5**	**368.37**	**-0.29**
**ZINC00794058**	**82.85**	**32**	**437.47**	**7**	**1**	**0**	**4**	**386.57**	**0.03**
**ZINC00686337**	**82.50**	**32**	**441.43**	**7**	**1**	**0**	**4**	**374.94**	**0.07**
**ZINC09550295**	**82.85**	**33**	**471.92**	**7**	**1**	**0**	**5**	**400.34**	**0.03**
**Gemifloxacin**	**123.06**	**28**	**389.39**	**9**	**3**	**0**	**5**	**329.02**	**0.67**

[a] Tpsa (topological polar surface area), [b] Molecular weight, [c] number of H-bond donors, [d] number of H-bond acceptors, [e] Number of rotatable bonds

Table S2. Physicochemical parameters and drug-likeness properties of the hit compounds obtained from the Osiris program.

**Compounds**	**clogP**	**solubility**	**Drug-like score**	**Overall drug score**
**ZINC77906236**	**2.83**	**-4.89**	**1.80**	**0.58**
**ZINC03114214**	**5.40**	**-8.42**	**-6.26**	**0.15**
**ZINC09550296**	**3.02**	**-5.47**	**6.39**	**0.51**
**ZINC77906466**	**2.54**	**-4.84**	**1.42**	**0.57**
**ZINC01958447**	**5.32**	**-8.47**	**-4.62**	**0.15**
**ZINC09751390**	**2.33**	**-4.88**	**6.49**	**0.56**
**ZINC01269201**	**1.49**	**-4.69**	**0.45**	**0.51**
**ZINC21985520**	**1.71**	**-4.29**	**0.68**	**0.55**
**ZINC09751395**	**2.33**	**-4.88**	**6.49**	**0.56**
**ZINC02280291**	**2.45**	**-4.80**	**1.70**	**0.31**
**ZINC01440193**	**2.69**	**-4.83**	**2.85**	**0.32**
**ZINC01127091**	**2.03**	**-4.52**	**3.84**	**0.50**
**ZINC72332562**	**3.61**	**-7.15**	**1.84**	**0.39**
**ZINC00794058**	**2.51**	**-4.29**	**4.50**	**0.67**
**ZINC00686337**	**2.27**	**-4.26**	**5.58**	**0.68**
**ZINC09550295**	**2.77**	**-4.65**	**6.20**	**0.60**
**Gemifloxacin**	**-1.63**	**-4.09**	**3.09**	**0.75**

Table S3. Calculated SAR properties of the dataset obtained from the Molinspiration program.

Molecules	TPSA[a]	natoms	M.Wt[b]	nON[c]	nOHNH[d]	n rotb[e]	Volume	Enzyme inhibitor
7a	109.08	26	364.33	8	2	5	303.06	0.22
7b	99.84	25	348.33	7	2	5	294.07	0.24
7c	99.84	26	362.36	7	2	5	310.88	0.24
7d	99.84	27	376.38	7	2	5	327.46	0.19
7e	126.15	31	434.42	9	2	8	372.23	0.09
7f	128.52	35	478.48	9	3	6	407.13	0.21
7g	111.87	26	363.35	8	3	5	306.48	0.24
7h	103.08	27	377.37	8	2	5	323.42	0.22
7i	123.31	29	407.40	9	3	7	348.48	0.19
7j	128.86	32	441.42	10	2	6	369.95	0.21
7k	103.08	33	453.47	8	2	7	395.07	0.17
7l	103.08	32	439.44	8	2	6	378.26	0.15
7n	99.84	27	376.38	7	2	5	327.68	0.23
7o	154.96	30	420.40	10	5	6	353.57	0.13
7p	137.14	29	406.37	9	3	6	337.90	0.19

[a] Tpsa (topological polar surface area), [b] Molecular weight, [c] number of H-bond donors, [d] number of H-bond acceptors, [e] Number of rotatable bonds

Table S4. Physicochemical parameters and drug-likeness properties of the dataset obtained from the Osiris program.

Molecules	clogP	Solubility	Drug likeness Score	Overall drug Score
7a	-0.72	-2.47	-5.52	0.45
7b	0.10	-3.09	-4.91	0.44
7c	0.44	-3.36	-6.41	0.43
7d	0.71	-3.52	-3.32	0.43
7e	1.02	-3.98	-4.94	0.37
7f	1.51	-4.08	-2.27	0.37
7g	-2.93	-2.33	-4.06	0.46
7h	-1.54	-1.97	-0.53	0.61
7i	-2.06	-1.76	-1.14	0.54
7j	-0.31	-3.08	-1.41	0.48
7k	-0.12	-3.29	-1.45	0.46
7l	0.89	-3.81	-1.02	0.47
7n	0.79	-3.63	-8.91	0.41
7o	-1.72	-3.34	-7.91	0.24
7p	-0.56	-2.99	-4.12	0.43

Table S5. SAR properties of designed pharmacophore analogs.

Molecules	TPSA[a]	natoms	M.Wt[b]	nON[c]	nOHNH[d]	n violations	n rotb[e]	volume	Enzyme inhibitor
**Mol1**	**62.54**	**24**	**332.38**	**5**	**1**	**0**	**3**	**300.22**	**0.13**
**Mol2**	**62.54**	**23**	**318.34**	**5**	**1**	**0**	**3**	**283.63**	**0.17**
**Mol3**	**88.56**	**24**	**333.36**	**6**	**3**	**0**	**3**	**294.95**	**0.27**
**Mol4**	**65.78**	**25**	**347.39**	**6**	**1**	**0**	**3**	**312.76**	**0.21**
**Mol5**	**65.78**	**23**	**319.34**	**6**	**1**	**0**	**2**	**279.37**	**0.16**
**Mol6**	**74.57**	**22**	**305.31**	**6**	**2**	**0**	**2**	**262.43**	**0.19**
**Mol7**	**86.01**	**25**	**349.36**	**7**	**2**	**0**	**4**	**304.43**	**0.14**
**Mol8**	**86.01**	**26**	**363.39**	**7**	**2**	**0**	**5**	**321.24**	**0.13**
**Mol9**	**86.01**	**27**	**377.42**	**7**	**2**	**0**	**5**	**337.82**	**0.18**
**Mol10**	**65.78**	**29**	**395.43**	**6**	**1**	**0**	**4**	**351.02**	**0.12**
**Mol11**	**65.78**	**30**	**409.46**	**6**	**1**	**0**	**5**	**367.82**	**0.11**
**Mol12**	**65.78**	**31**	**423.49**	**6**	**1**	**0**	**5**	**384.41**	**0.15**
**Mol13**	**65.78**	**30**	**409.46**	**6**	**1**	**0**	**4**	**367.61**	**0.14**
**Mol14**	**65.78**	**28**	**381.41**	**6**	**1**	**0**	**3**	**334.22**	**0.10**
**Mol15**	**65.78**	**29**	**395.43**	**6**	**1**	**0**	**4**	**351.02**	**0.09**
**Mol16**	**82.85**	**24**	**333.32**	**7**	**1**	**0**	**2**	**281.80**	**0.21**
**Mol17**	**82.85**	**25**	**347.35**	**7**	**1**	**0**	**2**	**298.36**	**0.09**
**Mol18**	**82.85**	**26**	**361.37**	**7**	**1**	**0**	**3**	**315.16**	**0.08**
**Mol19**	**82.85**	**27**	**375.40**	**7**	**1**	**0**	**3**	**331.75**	**0.14**
**Mol20**	**71.78**	**22**	**306.29**	**6**	**1**	**0**	**2**	**259.01**	**0.16**
**Mol21**	**71.78**	**23**	**320.32**	**6**	**1**	**0**	**3**	**275.81**	**0.14**
**Mol22**	**71.78**	**24**	**334.35**	**6**	**1**	**0**	**3**	**292.40**	**0.20**
**Mol23**	**65.78**	**24**	**329.40**	**6**	**1**	**0**	**3**	**307.83**	**0.21**
**Mol24**	**65.78**	**23**	**315.37**	**6**	**1**	**0**	**3**	**291.24**	**0.15**
**Mol25**	**65.78**	**22**	**301.35**	**6**	**1**	**0**	**2**	**274.44**	**0.17**
**Mol26**	**65.78**	**27**	**363.42**	**6**	**1**	**0**	**3**	**329.29**	**0.12**
**Mol27**	**65.78**	**28**	**377.44**	**6**	**1**	**0**	**4**	**346.09**	**0.11**
**Mol28**	**65.78**	**29**	**391.47**	**6**	**1**	**0**	**4**	**362.68**	**0.16**
**Mol29**	**65.78**	**28**	**377.44**	**6**	**1**	**0**	**4**	**346.09**	**0.12**
**Mol30**	**65.78**	**29**	**391.47**	**6**	**1**	**0**	**5**	**362.89**	**0.11**
**Mol31**	**65.78**	**30**	**405.50**	**6**	**1**	**0**	**5**	**379.48**	**0.16**
**Mol32**	**65.78**	**29**	**411.89**	**6**	**1**	**0**	**4**	**359.63**	**0.06**
**Mol33**	**65.78**	**30**	**425.92**	**6**	**1**	**0**	**5**	**376.43**	**0.05**
**Mol34**	**65.78**	**31**	**439.94**	**6**	**1**	**0**	**5**	**393.02**	**0.10**
**Mol35**	**65.78**	**25**	**363.85**	**6**	**1**	**0**	**3**	**321.37**	**0.14**
**Mol36**	**65.78**	**24**	**349.82**	**6**	**1**	**0**	**3**	**304.78**	**0.08**
**Mol37**	**120.15**	**27**	**393.78**	**9**	**2**	**0**	**4**	**317.64**	**0.20**
**Mol38**	**99.84**	**25**	**364.79**	**7**	**2**	**0**	**4**	**302.68**	**0.18**
**Mol39**	**99.84**	**24**	**350.76**	**7**	**2**	**0**	**4**	**285.88**	**0.18**
**Mol40**	**111.87**	**25**	**365.77**	**8**	**3**	**0**	**4**	**298.28**	**0.18**

[a] Tpsa (topological polar surface area), [b] Molecular weight, [c] number of H-bond donors, [d] number of H-bond acceptors, [e] Number of rotatable bonds

Table S6. Physicochemical parameters and drug-likeness properties of designed molecules.

Molecules	clogP	solubility	Drug-like score	Overall drug score
Mol1	1.99	-4.04	2.98	0.77
Mol2	1.72	-3.88	0.24	0.60
Mol3	-1.26	-3.56	2.69	0.82
Mol4	0.10	-2.87	5.33	0.88
Mol5	-0.67	-2.19	5.16	0.92
Mol6	-2.06	-2.56	2.27	0.87
Mol7	-1.19	-1.99	5.13	0.90
Mol8	-0.78	-2.99	5.09	0.89
Mol9	-0.42	-2.66	4.77	0.86
Mol10	0.75	-3.52	4.83	0.80
Mol11	1.16	-3.82	4.78	0.76
Mol12	1.52	-4.19	4.46	0.71
Mol13	2.52	-4.72	4.87	0.66
Mol14	1.76	-4.04	5.25	0.76
Mol15	2.16	-4.34	5.20	0.72
Mol16	-0.05	-2.64	2.84	0.87
Mol17	0.32	-2.48	5.01	0.89
Mol18	0.72	-2.78	5.01	0.87
Mol19	1.08	-3.15	4.39	0.83
Mol20	0.50	-2.69	0.80	0.77
Mol21	0.56	-2.99	0.77	0.75
Mol22	0.92	-3.37	0.47	0.69
Mol23	-0.00	-2.56	6.00	0.90
Mol24	-0.36	-2.18	6.34	0.92
Mol25	-0.77	-1.88	5.83	0.93
Mol26	1.65	-3.73	5.92	0.80
Mol27	2.06	-4.03	5.87	0.76
Mol28	2.42	-4.40	5.54	0.71
Mol29	0.65	-3.20	5.50	0.46
Mol30	1.06	-3.50	5.46	0.80
Mol31	1.42	-3.88	5.14	0.76
Mol32	1.26	-3.94	6.37	0.75
Mol33	1.66	-4.24	6.33	0.71
Mol34	2.02	-4.62	6.02	0.65
Mol35	0.60	-3.29	6.86	0.84
Mol36	0.25	-2.92	7.20	0.87
Mol37	-0.42	-2.84	-1.89	0.48
Mol38	0.95	-3.78	-4.83	0.41
Mol39	0.61	-3.51	-3.32	0.43
Mol40	-2.42	-2.75	-2.46	0.47

Table S7. Molecular docking results of designed pharmacophore analogs.

Molecules	Binding Energy (kcal/mol)	Fitness score	S(hb_ext)	S(vdw_ext)	S(vdw_int)
**Mol1**	**-8.5**	**59.31**	**6.99**	**48.60**	**-14.51**
**Mol2**	**-8.3**	**58.54**	**6.58**	**47.28**	**-13.05**
**Mol3**	**-8.0**	**60.55**	**6.20**	**48.58**	**-12.45**
**Mol4**	**-8.4**	**62.00**	**6.23**	**50.02**	**-13.00**
**Mol5**	**-8.1**	**53.87**	**1.07**	**46.97**	**-11.78**
**Mol6**	**-8.3**	**51.95**	**0.56**	**45.56**	**-11.25**
**Mol7**	**-8.3**	**52.96**	**0.88**	**49.70**	**-16.26**
**Mol8**	**-8.3**	**57.65**	**1.11**	**52.33**	**-15.41**
**Mol9**	**-8.5**	**60.84**	**7.52**	**53.22**	**-19.87**
**Mol10**	**-10.1**	**62.36**	**0.07**	**56.16**	**-14.92**
**Mol11**	**-10.1**	**68.02**	**0.58**	**58.93**	**-13.59**
**Mol12**	**-9.9**	**68.38**	**0.26**	**59.77**	**-14.07**
**Mol13**	**-9.4**	**52.61**	**0.21**	**49.92**	**-16.24**
**Mol14**	**-9.4**	**55.21**	**3.01**	**49.62**	**-16.04**
**Mol15**	**-9.3**	**60.75**	**1.45**	**53.43**	**-14.17**
**Mol16**	**-8.1**	**51.11**	**1.09**	**44.80**	**-11.58**
**Mol17**	**-8.7**	**51.07**	**8.00**	**46.07**	**-20.28**
**Mol18**	**-8.7**	**58.27**	**1.76**	**49.28**	**-11.24**
**Mol19**	**-8.7**	**56.41**	**0.39**	**49.64**	**-12.24**
**Mol20**	**-8.1**	**50.73**	**2.58**	**43.38**	**-11.49**
**Mol21**	**-8.1**	**54.85**	**0.55**	**48.26**	**-12.06**
**Mol22**	**-7.0**	**56.53**	**0.83**	**48.02**	**-10.32**
**Mol23**	**-8.5**	**57.55**	**0.67**	**50.31**	**-12.30**
**Mol24**	**-8.3**	**58.51**	**6.46**	**49.11**	**-15.48**
**Mol25**	**-8.3**	**51.82**	**1.52**	**45.78**	**-12.64**
**Mol26**	**-9.5**	**55.17**	**1.26**	**49.59**	**-14.28**
**Mol27**	**-9.4**	**53.98**	**0.85**	**49.30**	**-14.67**
**Mol28**	**-9.5**	**58.46**	**1.44**	**52.37**	**-15.00**
**Mol29**	**-9.8**	**60.04**	**0.04**	**53.84**	**-14.03**
**Mol30**	**-9.9**	**64.32**	**0.06**	**56.02**	**-12.77**
**Mol31**	**-10.1**	**67.54**	**1.32**	**57.95**	**-13.46**
**Mol32**	**-9.7**	**63.17**	**1.07**	**54.48**	**-12.82**
**Mol33**	**-9.5**	**60.41**	**0.53**	**52.46**	**-12.26**
**Mol34**	**-10.6**	**62.95**	**7.98**	**55.18**	**-21.91**
**Mol35**	**-8.3**	**54.59**	**6.57**	**47.05**	**-16.67**
**Mol36**	**-7.9**	**58.57**	**5.92**	**48.17**	**-13.50**
**Mol37**	**-9.0**	**54.26**	**0.49**	**49.00**	**-13.60**
**Mol38**	**-8.9**	**59.09**	**2.65**	**48.02**	**-9.59**
**Mol39**	**-9.0**	**53.39**	**1.13**	**49.68**	**-16.04**
**Mol40**	**-8.6**	**55.69**	**5.09**	**46.38**	**-13.16**

[a]Protein-Ligand H-bond Scores, [b]Protein-Ligand van der Waals scores, [c]Intramolecular van der Waals strain within the ligand

Table S8. Molecular structures and corresponding activities of the selected dataset.

Structures	Activity(μgr/mL)
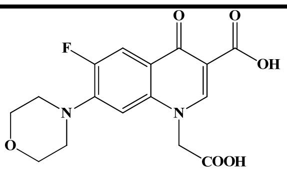 7a	37.5
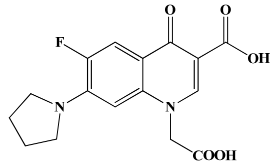 7b	37.5
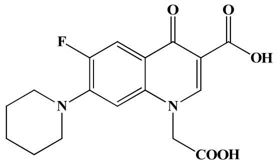 7c	150
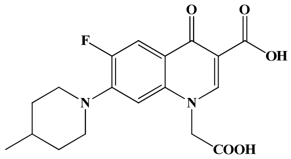 7d	150
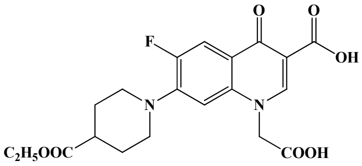 7e	75
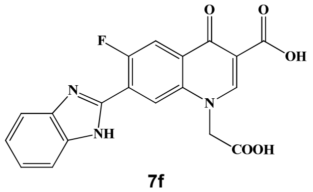 7f	150
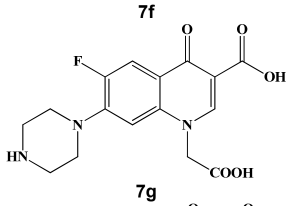 7g	37.5
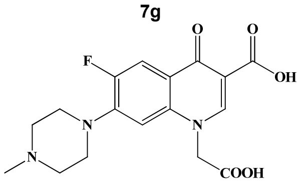 7h	150

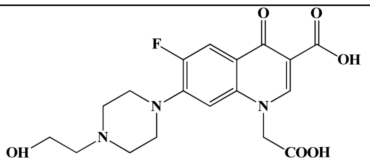 7i	150
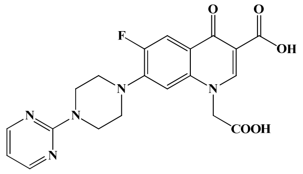 7j	150
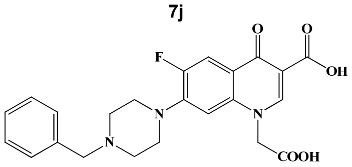 7k	150
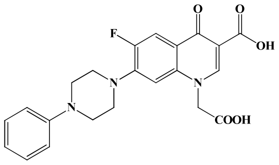 7l	150
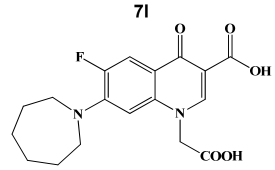 7n	150
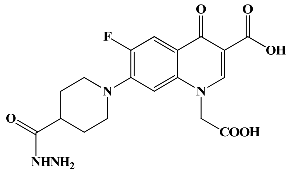 7o	150
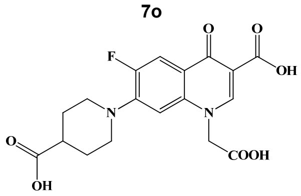 7p	75
